# Proteomic Analysis of Cardioembolic and Large Artery Atherosclerotic Clots Using Reverse Phase Protein Array Technology Reveals Key Cellular Interactions Within Clot Microenvironments

**DOI:** 10.7759/cureus.13499

**Published:** 2021-02-22

**Authors:** Mehdi Abbasi, Sean Fitzgerald, Jennifer Ayers-Ringler, Virginia Espina, Claudius Mueller, Sally Rucker, Ramanathan Kadirvel, David Kallmes, Waleed Brinjikji

**Affiliations:** 1 Radiology, Mayo Clinic, Rochester, USA; 2 Physiology, National University of Ireland Galway, Galway, IRL; 3 Center for Applied Proteomics and Molecular Medicine, George Mason University, Manassas, USA; 4 Neuroradiology, Mayo Clinic, Rochester, USA

**Keywords:** endocannabinoid, inflammation, phosphoprotein, proteomics, thrombus, stroke, thrombectomy

## Abstract

Thrombus characteristics are dependent on clot composition, but identification of the etiology based on histological analysis has proved inconclusive. Identification of proteomic signatures may help to differentiate between clots of different etiologies such as cardioembolic, large artery atherosclerotic, and other known etiologies, information that could enhance an individualized medicine approach to secondary stroke prevention. In this study, total protein extracts from cardioembolic (n=25) and large artery atherosclerotic (n=23) thrombus specimens were arrayed in quadruplicate on nitrocellulose slides and immunostained for 31 proteins using a Dako Autostainer (Agilent Technologies, Inc., Santa Clara, USA). We quantified 31 proteins involved in platelet and/or endothelial function, inflammation, oxidative stress, and metabolism. Pathway analysis showed more heterogeneity and protein network interactions in the cardioembolic clots but no specific correlations with clot etiology. Reverse-phase protein arrays are a powerful tool for assessing cellular interactions within the clot microenvironment and may enhance understanding of clot formation and origination. This tool could be further explored to help in identifying stroke etiology in large vessel occlusion patients with embolic stroke of an undetermined source.

## Introduction

Ischemic stroke is caused by a thromboembolic occlusion of cerebral arteries. Treatment is focused on fast and efficient removal of the occluding clot with either intravenous thrombolysis or with endovascular thrombectomy. Although the occluding clot is the primary target of treatment, little is known about its internal organization and inflammatory or cytokines that are involved in the formation or evolution of clots. Also, cellular pathways and cells governing the structure of clots has become a hot topic in the medical literature. A recent subgroup analysis from the Multicenter Randomized Clinical Trial of Endovascular Treatment for Acute Ischemic Stroke in the Netherlands (MR CLEAN) Registry has confirmed that thrombus computed tomography characteristics of cardioembolic (CE) stroke are distinct from those of non-CE stroke. Non-CE strokes were associated with the presence of hyperdense artery sign, a higher clot burden, a shift towards a more proximal thrombus location, and longer thrombi compared with a CE cause [1]. The differences in imaging characteristics between stroke etiologies are likely a result of differences in the composition of the occlusive thrombus resulting from dissimilarities in the clotting mechanisms. Histological analysis can reveal microscopic clot composition, but studies relating etiology to clot composition have thus far proved inconclusive [2]. Identification of proteomic signatures of thrombi from different stroke etiologies may prove invaluable in identifying the true etiology of the roughly 40% of ischemic stroke patients that have no known cause, which complicates effective secondary stroke prevention in these patients [3]. This study sought to investigate the proteomic signature of CE and large artery atherosclerotic (LAA) clots.

## Materials and methods

Patients

All patients underwent comprehensive neurovascular imaging for evaluation of the presence of carotid atherosclerotic disease, including CT angiography from the aorta to the intracranial arterial vasculature. All patients also underwent a standard cardiac evaluation, including a transthoracic echocardiogram (TTE)/transesophageal echocardiogram (TEE), electrocardiogram (EKG). In cases in which no abnormality was found on the initial cardiac workup, prolonged cardiac monitoring was performed using either a Holter monitor or implantable cardiac monitoring device. Patients were categorized according to Toast criteria [4]. The study was approved by the Mayo Clinic Institutional Review Board, and a waiver of consent was granted.

Specimens

Thrombus specimens were collected from patients undergoing mechanical thrombectomy at Mayo Clinic between October 2016 and July 2019 (Table 1). The CE (n=25) and LAA (n=23) thrombus specimens were fixed in 10% neutral buffered formalin for up to 24 hours and processed into paraffin blocks (FFPE). Two representative slides from each clot (3µm-thick sections, two-section on each slide) were stained with Martius Scarlett Blue stain. After regular histological staining, three 50μm sections were cut from each FFPE block, placed into a microcentrifuge tube, and stored at 4°C. Two tissue sections from each specimen were deparaffinized in the tube using two exchanges of xylene (three minutes at 50°C). The tissue sections were rehydrated in graded ethanol (100%, 95%, 70%, water with protease inhibitors (Roche cOmplete™ Mini​​​​​​), and lysed in protein lysis buffer (450μL tissue protein extraction reagent (Pierce T-PER™), 450μL tris-glycine 2X sodium dodecyl sulfate (SDS) sample buffer (Invitrogen™), and 100μL tris(2-carboxyethyl)phosphine (Pierce™ TCEP Solution, neutral pH, Bond-Breaker™​​​​​​​). Tissue lysates were heated at 95°C for eight minutes and stored at -80°C.

**Table 1:** Baseline characteristics of patients NIHSS - National Institutes of Health Stroke Scale; ICA - internal carotid artery; rTPA - recombinant tissue plasminogen activator; LAA - large artery atherosclerotic; TICI - thrombolysis in cerebral infarction

	LAA group, n (N=23)	Cardioembolic group, n (N=25)
Age (years), mean (±SD)	68 (±11)	69 (±13)
Gender (male)	15	12
NIHSS at admission, mean (±SD)	17 (±6)	17 (±7)
Diabetes	5	4
Hypertension	10	18
Dyslipidemia	12	15
Smoking	1	4
Occlusion location		
ICA	5	7
ICA terminus	2	4
M1	11	13
M2	2	3
M3	1	0
Vertebral	1	0
Basilar	1	0
P1	0	1
rTPA technique	10	13
Aspiration	16	17
Stentretriever	2	3
Combination	5	5
No of passes, mean (±SD)	2.2 (±1.3)	2 (±1)
Final TICI score (2b or higher)	21	23

Reverse phase protein arrays (RPPA)

**Figure 1:**
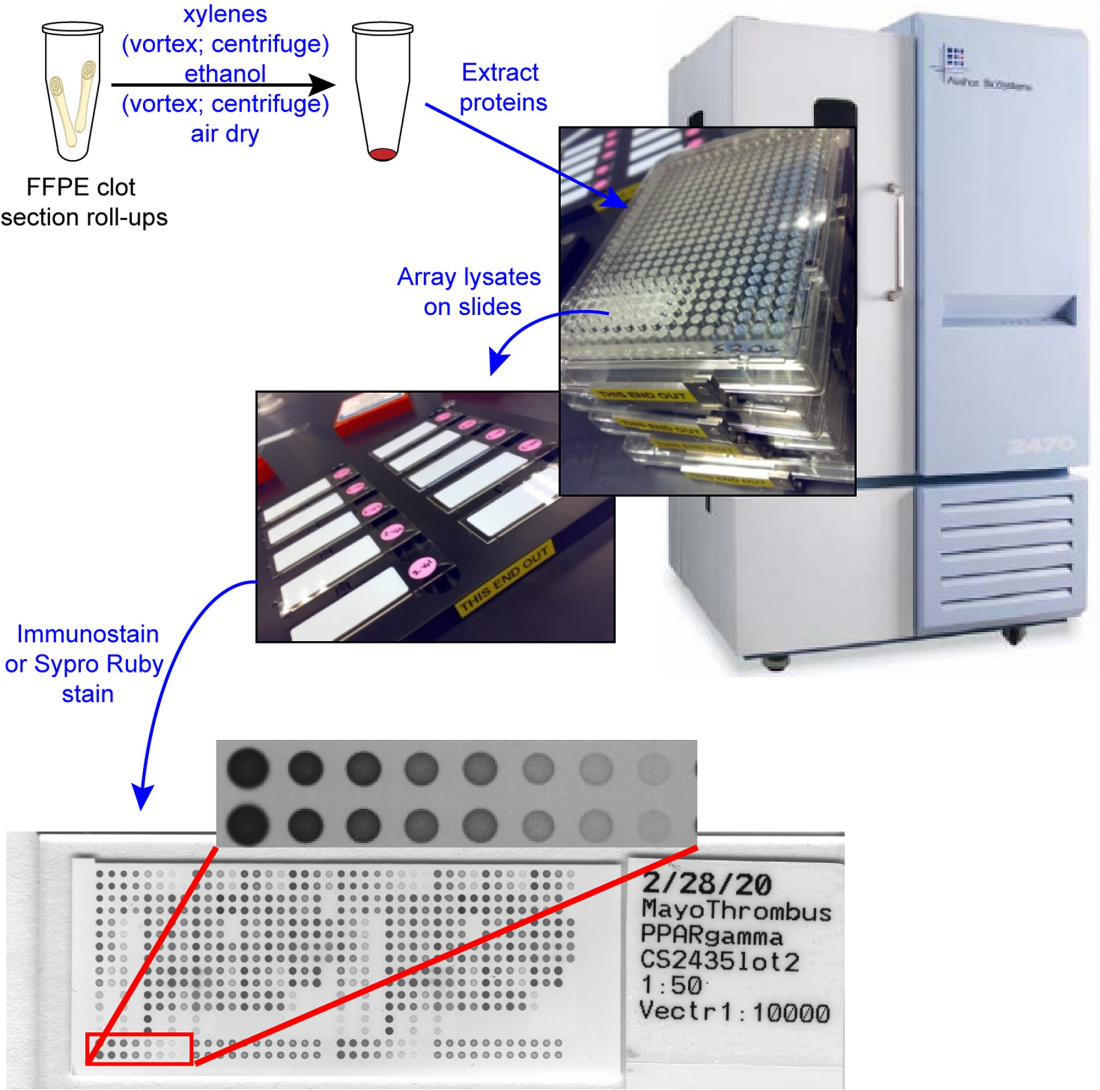
Proteomic workflow using formalin-fixed, paraffin-embedded (FFPE) thrombus specimens Proteins were extracted from FFPE clot specimens, arrayed on nitrocellulose-coated glass slides using a Quanterix 2470 arrayer, and immunostained, or stained for total protein on a Dako Autostainer.

Thrombus lysates were printed in technical quadruplicates onto nitrocellulose coated glass slides (ONCYTE® Avid, Grace Bio-Labs, Bend, USA), in serial 2-fold dilution curves, using a Quanterix™​ 2470 arrayer (Billerica, USA) equipped with 350μm solid pins (Figure 1). EB1 (BD 611546, Burkitt’s lymphoma), MOLT-4 (Santa Cruz Biotechnology sc-2233, acute lymphoblastic leukemia), normal umbilical vein tissue (Abcam ab29585), and Jurkat+Calyculin (Santa Cruz Biotechnology sc-2277, human acute T cell leukemia induced with Calyculin A cell lysates) were printed on each array as quality control samples. Bovine serum albumin (BSA) was printed in an 8 point, 2-fold dilution series, starting at 1.0 mg/mL as a calibration curve for total protein [5, 6]. The total protein within each array spot was determined using SYPRO® Ruby protein blot stain /Invitrogen™ Molecular Probes™) per manufacturer’s directions and scanned using a Cy3 laser (PowerScanner™, Tecan Group Ltd., Männedorf, Switzerland). Immunostaining for 31 proteins was performed on a Dako Autostainer per the manufacturer’s instructions (CSA kit, Dako, Agilent Technologies, Inc., Santa Clara, USA). Each slide was incubated with a single primary antibody at room temperature for 30 minutes (Table 2). Antibody specificity was confirmed by Western blotting as previously described [7]. The negative control slide was incubated with antibody diluent only. The secondary antibody was goat anti-rabbit immunoglobulin G (IgG) H+L (1:10,000; Vector Labs, Burlingame, USA). Signal detection was amplified via horseradish peroxidase mediated biotinyl tyramide deposition with chromogenic detection (diaminobenzidine) per manufacturer’s instructions (Dako, Agilent Technologies, Inc., Santa Clara, USA). Arrays were scanned at 600dpi on a flatbed scanner (PowerLook, UMAX®, Dallas, USA). Spot (pixel) intensity was analyzed using ImageQuant™​ ver. 5.2 (GE Healthcare, Chicago, USA), with mean local area background subtraction. Signal intensity for each spot was calculated using the freely available data reduction algorithm (RAS ver. 16) [5]. Spot intensities were normalized to total protein/spot.

**Table 2:** Protein names and antibody information for reverse phase protein arrays RPPA - reverse phase protein arrays

RPPA protein	Full name	Company	Catalog #	Dilution
AcCoA Ser79	Acetyl-CoA carboxylase phospho Ser79	Cell Signaling Technology	3661	1:1000
Androgen receptor	Androgen receptor	Cell Signaling Technology	5153	1:100
Arginase-1	Arginase-1	Cell Signaling Technology	9819	1:100
Biliverdin reductase	Biliverdin reductase	Assay Designs	0SA-400	1:750
CD234/DARC	Duffy antigen chemokine receptor	Pierce	PA5-18424	1:50
CD63	CD63	Abcam	ab134045	1:1000
CD45	Protein tyrosine phosphatase receptor type C	BD	610265	1:200
CD79a	B-cell antigen receptor complex-associated protein alpha chain	Cell Signaling Technology	13333	1:100
Cannabinoid receptor I (CNR1, CB1)	Cannabinoid receptor 1	Pierce	PA1-745	1:50
Cannabinoid receptor II (CNR2, CB2)	Cannabinoid receptor 2	Pierce	PA1-746A	1:100
Collagen I	Collagen I	Santa Cruz Biotechnology	sc-80760	1:50
Collagen IV	Collagen IV	Dako	M0785	1:50
DAG-lipase beta	Diacylglycerol-lipase beta	Cell Signaling Technology	12574	1:100
eNOS Ser177	Endothelial nitric oxide synthase phospho Ser177	Cell Signaling Technology	9571	1:50
Galectin-3	Galectin-3	Cell Signaling Technology	87985	1:300
GRK2	G-protein coupled receptor kinase 2/ β-adrenergic receptor kinase 1 (β-ARK1)	Cell Signaling Technology	3982	1:250
IL-6	Interleukin-6	BioVision	5143-100	1:100
IL-8	Interleukin-8	Abcam	ab7747	1:200
IL-10	Interleukin-10	Abcam	ab52909	1:2000
IL-11	Interleukin-11	Santa Cruz Biotechnology	sc-7924	1:500
Neutrophil elastase	Neutrophil elastase	Abcam	ab68672	1:100
PDK-1 Ser241	Phosphoinositide-dependent protein kinase 1 phospho Ser241	Cell Signaling Technology	3061	1:200
PKC alpha Ser657	Protein kinase C α phospho Ser657	Upstate	06-822	1:1000
PKC alpha/beta Thr638/641	Protein kinase C α/β phospho Thr638-641	Cell Signaling Technology	9375	1:200
PLC gamma Y783	Phosphoinositide-specific phospholipase C phospho Tyr783	Cell Signaling Technology	2821	1:100
PP2A	Protein phosphatase 2A	Cell Signaling Technology	2039	1:1000
PPARgamma	Peroxisome proliferator-activated receptor-gamma	Cell Signaling Technology	2435	1:50
Serotonin	Serotonin (5-hydroxytryptophan, 5-HT)	Sigma	S5545	1:1000
Syk Y525/526	Spleen tyrosine kinase phospho525/526	Cell Signaling Technology	2711	1:50
TGF-β	Transforming growth factor β	Cell Signaling Technology	3709	1:2000
vWF	von Willebrand factor	Cell Signaling Technology	65707	1:100

Statistical methods 

Normality was assessed using the Shapiro-Wilk test. Wilcoxon signed-rank sum pairwise comparisons were used for non-normally distributed data with p≤0.05 considered significant. Data was standardized (z-score) for each protein. Two-way, hierarchical cluster analysis (Euclidean distance, Ward method), correlograms, and Spearman’s rho non-parametric correlations were performed in R (R Foundation for Statistical Computing, Vienna, Austria). Spearman’s rho non-parametric correlations were used to compare pairs of protein endpoints (rho ≥0.85 and p≤0.001 were considered significant). Network diagrams were created in Cytoscape ver. 3.8.0 [8].

## Results

Histological composition of LAA and cardioembolic clots

**Figure 2:**
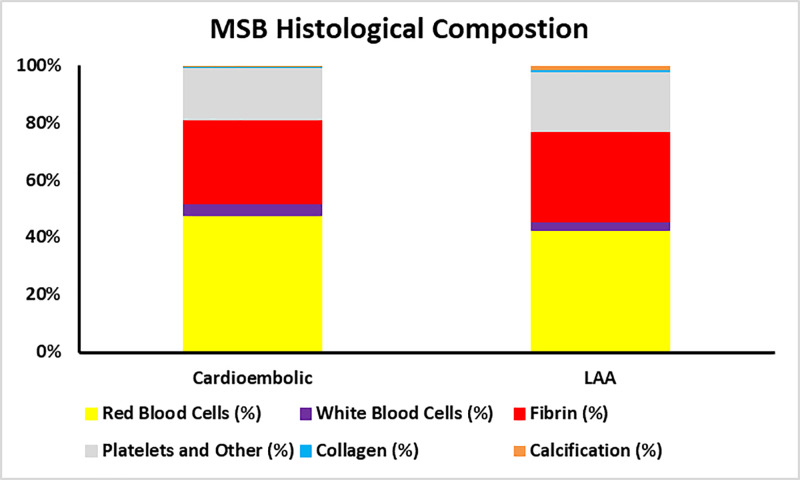
Histological clot composition of cardioembolic and LAA clots The mean histological clot composition (%) of clot component, as determined by MSB staining represented as the percentage of the total. MSB - Martius scarlet blue; LAA - large artery atherosclerotic

The Martius scarlet blue (MSB) histological analysis revealed that there were slight differences in the histological clot composition between the LAA and cardioembolic clots (Figure 2). Cardioembolic clots had a higher proportion of red blood cells (RBCs; 47.67% vs. 42.58%) and white blood cells (WBCs; 4.22% vs. 3.12%) compared to LAA clots. On the other hand, LAA clots had a slightly larger proportion of fibrin (31.31% vs. 29.19%) and platelets/other (20.81% vs. 18.21%). Collagen (0.10% vs. 0.59%) and calcification (0.61% vs. 1.59%) are minor components of both cardioembolic and LAA clots respectively.

Proteomic heterogeneity exists within classes of thrombus specimens.

**Figure 3:**
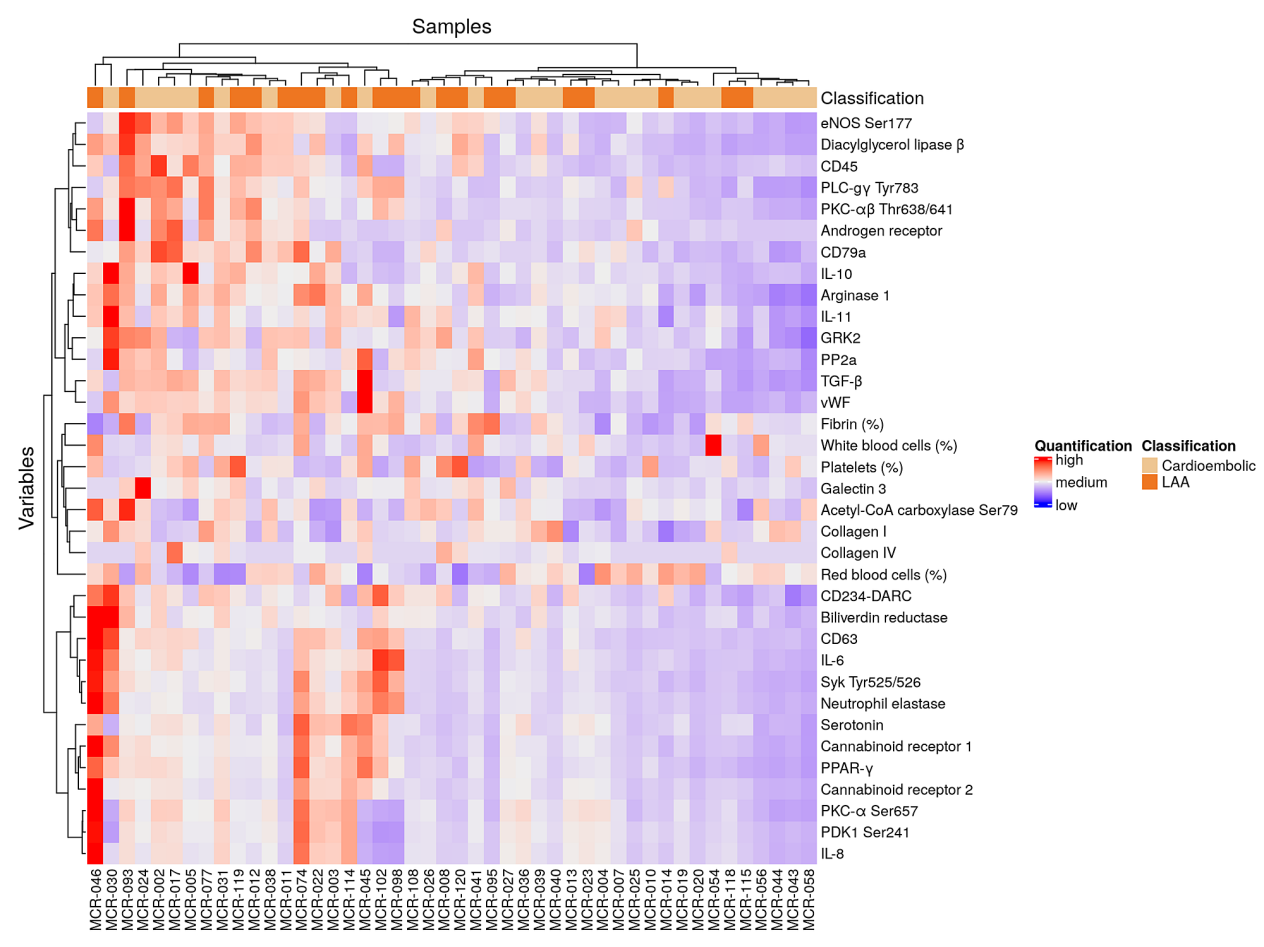
Proteomic heterogeneity exists within cardioembolic and large artery atherosclerotic thrombus specimens Unsupervised hierarchical two-way clustering of the entire cohort of thrombus specimens revealed two heterogeneous clusters of specimens (columns) that contained both CE and LAA thrombus specimens (n=25 CE, n=21 LAA, Euclidean distance, Ward method). The 'variables' dendrogram on the y-axis indicates the relationships between the expressions of all variables (rows) for all thrombus specimens. The 'variables' concentrations are depicted using a color scale from red (high), to white, to blue (low). LAA - large artery atherosclerotic; CE - cardioembolic

Data for two specimens in the LAA group were not used for hierarchical clustering and network analyses due to missing data (poor precision between technical replicates) for CD63, CD45, and collagen IV. Unsupervised two-way hierarchical clustering revealed two primary clusters of specimens; however, each cluster showed heterogeneity in relation to the etiology of the clot (Figure 3). The protein levels for the 31 proteins in our study could not clearly discriminate CE and LAA thrombus types.

Cardioembolic clots possess diverse protein signaling interactions

A pairwise protein linkage analysis was conducted using Spearman's rho non-parametric correlation to map inter-related signaling kinases. Fifty strong protein linkages were noted for the CE (rho≥0.85, p<0.001), while only 18 strong linkages were noted for the LAA group (Table 3). No significant negative protein interactions were noted for either group.

**Table 3:** Spearman rho non-parametric pairwise protein correlations quantified in cardioembolic (CE) and large artery atherosclerotic (LAA) thrombus specimens show extensive network linkages in CE but not LAA thrombus specimens JMP® Statistical Software ver. 5.1, SAS Institute Inc., Cary, USA

CE protein variable 1	CE protein variable 2	Spearman rho_CE (p<0.001)	LAA protein variable 1	LAA protein variable 2	Spearman rho_ LAA (p<0.001)
Arginase-1	IL-10	0.88			
Arginase-1	IL-6	0.87			
Arginase-1	IL-11	0.86			
Cannabinoid Rec 1	PKCaß Thr 638/641	0.90			
Cannabinoid Rec 1	CD234 (DARC)	0.87			
Cannabinoid Rec 2	Cannabinoid Rec 1	0.94	Cannabinoid Rec 2	Cannabinoid Rec 1	0.93
Cannabinoid Rec 2	Serotonin	0.88			
Cannabinoid Rec 2	PKCaß Thr 638/641	0.85			
CD63	Cannabinoid Rec 1	0.95			
CD63	Cannabinoid Rec 2	0.90			
eNOS Ser177	DAG Lipase Beta	0.89			
IL-10	Biliverdin Reductase	0.85			
IL-6	Cannabinoid Rec 1	0.97			
IL-6	CD63	0.96	IL-6	CD63	0.89
IL-6	Cannabinoid Rec 2	0.95			
IL-6	PKCaß Thr 638/641	0.88			
IL-6	CD234 (DARC)	0.88			
IL-8	PKCa Ser657	0.96	IL-8	PKCa Ser657	0.99
Neutrophil Elastase	IL-6	0.98	Neutrophil Elastase	IL-6	0.94
Neutrophil Elastase	Cannabinoid Rec 1	0.97	Neutrophil Elastase	Cannabinoid Rec 1	0.89
Neutrophil Elastase	Cannabinoid Rec 2	0.96	Neutrophil Elastase	Cannabinoid Rec 2	0.88
Neutrophil Elastase	CD63	0.94			
Neutrophil Elastase	CD234 (DARC)	0.90			
Neutrophil Elastase	PKCaß Thr 638/641	0.87			
PDK1 Ser241	PKCa Ser657	0.98	PDK1 Ser241	PKCa Ser657	0.99
PDK1 Ser241	IL-8	0.95	PDK1 Ser241	IL-8	0.98
PLCgamma Tyr783	PKCaß Thr 638/641	0.94			
PPARgamma	Neutrophil Elastase	0.98	PPARgamma	Neutrophil Elastase	0.91
PPARgamma	IL-6	0.98	PPARgamma	IL-6	0.89
PPARgamma	Cannabinoid Rec 1	0.98	PPARgamma	Cannabinoid Rec 1	0.95
PPARgamma	Syk Tyr525/526	0.97	PPARgamma	Syk Tyr525/526	0.93
PPARgamma	Cannabinoid Rec 2	0.95	PPARgamma	Cannabinoid Rec 2	0.86
PPARgamma	CD63	0.94			
PPARgamma	vWF	0.90			
PPARgamma	PKCaß Thr 638/641	0.89			
PPARgamma	CD234 (DARC)	0.88			
PPARgamma	Arginase-1	0.85			
Syk Tyr525/526	Cannabinoid Rec 1	0.97	Syk Tyr525/526	Cannabinoid Rec 1	0.91
Syk Tyr525/526	IL-6	0.96	Syk Tyr525/526	IL-6	0.92
Syk Tyr525/526	Neutrophil Elastase	0.95	Syk Tyr525/526	Neutrophil Elastase	0.96
Syk Tyr525/526	Cannabinoid Rec 2	0.91	Syk Tyr525/526	Cannabinoid Rec 2	0.86
Syk Tyr525/526	PKCaß Thr 638/641	0.91			
Syk Tyr525/526	CD63	0.91	Syk Tyr525/526	CD63	0.85
Syk Tyr525/526	CD234 (DARC)	0.86			
Syk Tyr525/526	DAG Lipase Beta	0.86			
vWF	IL-6	0.92			
vWF	Syk Tyr525/526	0.91			
vWF	CD63	0.90			
vWF	Cannabinoid Rec 1	0.90			
vWF	Neutrophil Elastase	0.85			

**Figure 4:**
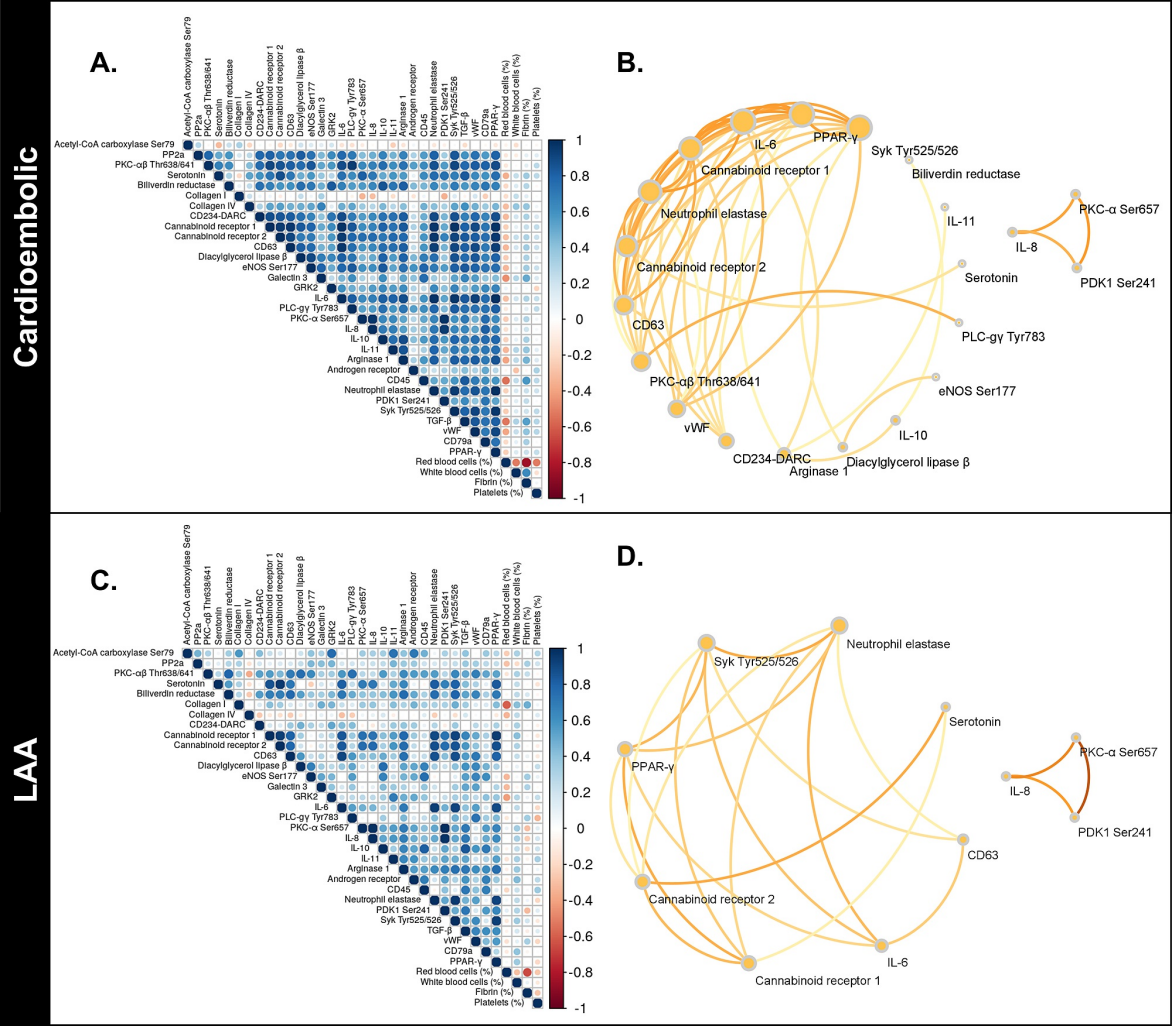
Protein interconnections and network signaling differ between thrombus etiology A correlogram of CE thrombus specimens reveals numerous, strong protein-protein interconnections, shown by the dark blue dots (A). Diverse protein network signaling predominates in CE thrombus specimens (B). In contrast, LAA clots show very few protein-protein interactions (C), with sparse network interconnections (D). LAA - large artery atherosclerotic; CE - cardioembolic

The protein linkages (correlations) in CE clot specimens were diverse and more abundant compared to LAA clot specimens (Figure 4). Predominant linkages between PPAR-gamma and arginase-1, CD63, CD234, PKCαβ Thr 638/641, and von Willebrand factor (vWF) were found in CE clots compared to LAA clots (Table 3). Multiple protein linkages within inflammatory/immune cell proteins arginase-1, neutrophil elastase, CD234, and CD63 were found in CE clot specimens. In contrast, the LAA clots did not have any unique protein-protein correlations compared to the CE clots and lacked significant vWF protein linkages.

## Discussion

Our study advances the knowledge of the human cerebrovascular thrombi composition by focusing on the proteomic analysis of cardioembolic and atherothrombotic thrombi. Our results support the general notion that direct analysis of the clots could help in the unveiling, in the future, biomarkers which potentially could assist in stroke diagnosis and differentiating stroke etiologies. Our study comparing the proteomic signatures of CE versus LAA clots demonstrated a number of interesting findings. We found predominant linkages between PPAR-gamma and arginase-1, CD63, CD234, PKCαβ Thr 638/641, and von Willebrand Factor (vWF) in CE clots suggesting that platelet signaling dominates in CE clots compared to LAA clots. The multiple protein linkages within inflammatory/immune cell proteins lend additional support to the observation of platelet-immune cell cross-talk in CE clot specimens, supported by the white blood cell enrichment seen in the histological analysis of CE emboli in this and previous studies [9]. Proteomic signaling does not necessarily correspond with cellular proportions on histological staining, but it does reflect activated pathways and intra- and inter-cellular communications present in the occlusive clot, influenced by the etiology. Detailed analysis including individual patient histories and medications will likely demonstrate strong correlations with certain protein networks, a study we plan to perform with the larger sample currently under investigation.

The widespread use of mechanical thrombectomy devices has resulted in the availability of clot material for histopathological and proteomic analysis. Studies relating histological clot composition to etiology have thus far proved inconclusive [2]. Identifying stroke etiology in this population is very important due to the fact that up to 30% of large vessel occlusions (LVO) patients do not have a stroke source identified, which complicates secondary stroke prevention strategies. Secondary stroke prevention in this population is essential in preventing recurrent large vessel occlusion, an occurrence that can have a devastating effect on the patient [9]. RPPAs are a powerful tool for assessing proteomic interactions within the cellular microenvironment and may enhance our understanding of the clot formation process in various etiologies, which is why we sought to investigate the proteomic signatures of thrombi from LAA and cardiac etiologies.

Previous proteomics studies have provided some insight, but much more needs to be explored to identify pathways that could aid in differentiating stroke etiology. A previous proteomic study found that two inflammation proteins (integrin alpha-M and mitochondrial superoxide dismutase) are associated with high blood low-density lipoprotein (LDL) [10]. In another study, these two proteins failed to differentiate between cardioembolic vs. atherosclerotic etiology [11]. Also, another study analyzed four thrombi, with ~1,600 proteins identified [12]. To identify proteomic signatures that can help differentiate between clots of different etiologies such as cardiac and LAA, 31 proteins involved in platelet and/or endothelial function, inflammation, oxidative stress, and metabolism were quantified using reverse phase protein arrays (RPPA) [13-15]. Unsupervised two-way hierarchical clustering revealed two primary clusters of specimens, but despite the difference in etiology between the two thrombus types, the resulting proteomic clusters were quite heterogeneous within each group. However, we did find that platelet signaling dominates in CE clots compared to LAA clots and hope to explore this hypothesis further in larger cohort studies.

Protein signaling networks comprise a series of interconnected proteins and their post-translationally modified forms [6, 16], which are generally organized within specific biological processes, such as inflammation or endothelial integrity. Crosstalk between pathways provides redundancy and resiliency within the cellular microenvironment. Mapping these signaling networks provides functional insights into metabolic processes [13, 16, 17]. The diverse protein signaling interactions found in the clots were determined primarily by analysis of protein phosphorylation cascades. Phosphorylation events are transient functional indicators of active kinase signaling pathways. Specific phosphorylation sites, at any point in time, define the functional character of protein-protein interactions. A set of interacting proteins within an active pathway will tend to be simultaneously phosphorylated in the signaling cascade. Thus, for a given set of patient observations over a disease cohort, correlations among individual protein pairs imply active interactions in a linked pathway [6, 18].

## Conclusions

While we demonstrated common protein interactions in thromboembolic disease, consisting of immune, endocannabinoid, and metabolism proteins, CE protein signaling networks are comprised of complex interactions across biological pathways and differ from those seen in LAA patients. Our findings suggest that RPPA of retrieved thrombi in large vessel occlusion could be a promising tool in determining stroke etiology. However, larger studies are needed.
